# Physiological Dysregulation, Frailty, and Impacts on Adverse Health and Functional Outcomes

**DOI:** 10.3389/fmed.2021.751022

**Published:** 2021-10-22

**Authors:** Yanxia Lu, Xinyi Gwee, Denise Q. L. Chua, Crystal T. Y. Tan, Keng Bee Yap, Anis Larbi, Tze Pin Ng

**Affiliations:** ^1^Department of Medical Psychology and Ethics, School of Basic Medical Sciences, Cheeloo College of Medicine, Shandong University, Jinan, China; ^2^Gerontology Research Programme, Department of Psychological Medicine, National University Health System, Yong Loo Lin School of Medicine, National University of Singapore, Singapore, Singapore; ^3^Biology of Aging Laboratory, Singapore Immunology Network (SIgN), Agency for Science, Technology and Research (A^*^STAR), Singapore, Singapore; ^4^Department of Geriatric Medicine, Ng Teng Fong Hospital, Singapore, Singapore; ^5^Geriatrics Division, Department of Medicine, Research Center on Aging, University of Sherbrooke, Sherbrooke, QC, Canada

**Keywords:** aging, allostatic load, frailty, endo-phenotype, mortality

## Abstract

**Background:** Multi-system physiological dysregulation (PD) may represent a biological endo-phenotype of clinical frailty. We investigated the co-occurrence of PD with physical frailty and its contributions to the known impact of frailty on adverse health outcomes.

**Methods:** Data of 2,725 participants from the Singapore Longitudinal Aging Studies (SLAS-2), included baseline measures of physical frailty and PD derived from Mahalanobis distance (Dm) value of 23 blood biomarkers. We analyzed their concurrent association and their impacts on 9-year mortality, MMSE cognition, GDS depression, number of medications, disability, and hospitalization at baseline and follow up (mean 4.5 years).

**Results:** Global PD (Log_10_Dm, mean = 1.24, *SD* = 0.24) was significantly but weakly associated with pre-frailty-and-frailty. Controlling for age, sex and education, pre-frailty-and-frailty (HR = 2.12, 95% CI = 1.51–3.00) and PD (HR = 3.88, 95% CI = 2.15–6.98) predicted mortality. Together in the same model, mortality HR associated with pre-frailty-and-frailty (HR = 1.83, 95% CI = 1.22–2.73) and PD (HR = 3.06, 95% CI = 1.60–5.85) were reduced after additionally adding global PD to the prediction model. The predictive accuracy for mortality were both approximately the same (PD: AUC = 0.62, frailty: AUC = 0.64), but AUC was significantly increased to 0.68 when combined (*p* < 0.001). Taken into account in the same model, frailty remained significantly associated with all health and functional outcomes, and PD was significantly associated with only MMSE, disability and medications used. In secondary analyses, there were mixed associations of system-specific PDs with frailty and different adverse outcomes.

**Conclusions:** Co-existing PD and physical frailty independently predict mortality and functional and health outcomes, with increased predictive accuracy when combined. PD appears to be a valid representation of a biological endo-phenotype of frailty, and the potential utility of such subclinical measures of frailty could be further studied.

## Introduction

Frailty is highly prevalent in old age. Numerous studies have shown that it strongly predicts adverse health outcomes, including complications, disability, mortality, hospitalizations, and institutionalization (1, 2) in both community-living older adults and hospitalized patient populations with specific chronic diseases. A conceptual definition of frailty is a diminished capacity (or physiological reserve) to adequately compensate for the effects of stressors, leading to an increased vulnerability to develop chronic diseases and adverse functional, health, and mortality outcomes (1). Operationally, frailty may be defined in terms of a multi-dimensional (physical, cognitive, mental, and social) construct (3, 4) or a physical frailty construct (5).

It is currently theorized that underpinning the development of frailty and age-related diseases in older people is a breakdown with age in the capacity to maintain physiological homeostasis via a complex regulatory network of factors, pathways, or processes which operate independently or inter-dependently in different physiological systems (6–12). Based on empirical observations, there is prima facie evidence that physiological dysregulation (PD) across multiple systems may appropriately represent the underlying biological substrate or endo-phenotype of the clinical frailty syndrome (13–16).

Physiological dysregulation may be measured using deviations of multiple molecular or cellular biomarker measurements from their normal ranges to derive a multivariate statistical distance (Mahalanobis distance, Dm), as proposed by Cohen and other investigators (8, 10, 17, 18). The Dm represents how aberrant an individual's profile is relative to all other individuals in the population, with greater distance indicating more dysregulation. In support of its representation as a biological endo-phenotype of frailty, the Dm measure of physiological dysregulation (10) has been shown to increase with age within individuals and predict mortality controlling for age (10, 19–21). Studies also show that PD predicts the onset of frailty (13–15). However, more empirical evidence is needed to elucidate many aspects of the complex relationships between PD, frailty and consequential adverse health and functional outcomes.

Several studies investigating the simultaneous effects of PD and frailty on mortality outcomes have reported that they independently predict survival (15, 16), and show as well that PD appears to be a stronger predictor of survival. Few studies have investigated the impacts of PD and frailty with respect to other known adverse health events preceding death. These include adverse functional outcomes such as cognitive impairment and depression (so-called “functional aging”) and adverse health outcomes such as polypharmacy, which may either precede or follow physical frailty. Other adverse health outcomes which are clearly consequences of frailty such as disability, hospitalization and institutionalization may at the same time aggravate frailty prior to death. Understanding the links between PD, frailty and different adverse health events provides deeper insights into the effects of PD underlying frailty in perpetuating the development of adverse health and functional and mortality outcomes in older adults.

Diverse biomarkers of inflammation, immune function, sympathetic nervous system function, endocrine system function, and cardiovascular, and metabolic function (22–27) are known to be implicated in the pathophysiology of frailty. Physiological dysregulation involving specific physiological systems may thus underlie physical frailty. Indeed, some studies show that multiple system-specific measures of PD predict diabetes, heart disease, and number of chronic diseases as well as overall mortality and frailty, with varying acuity beyond the effect of chronological age (19, 21).

In the present study, we investigated the association between PD and the physical phenotype of frailty, and their impacts on cognitive function and depressive symptoms, number of drug use, disability, hospitalization, and mortality. Specifically, we examined the extent to which global PD mediated the link between frailty and its associated functional and health and mortality outcomes. In secondary analyses, we explored the differential effects of system-specific PD and frailty on these adverse outcomes.

## Methods

### Study Design and Participants

The study participants were from the second recruitment wave cohort of the Singapore Longitudinal Aging Studies (SLAS-2) (28), a population-based prospective cohort study of aging and health transition among community-dwelling Chinese older adults aged 55 years and above. As described in previous publications (28), between March 2008 and November 2013, a total of 3,270 participants were interviewed. The extensive range of baseline data collected through questionnaire interviews, clinical and performance-based testing and examinations, and blood sampling included psychosocial, lifestyle and behavior, medical, biological, physiological, diet and nutrition, physical and neurocognitive functioning, and health status variables. The present study involved 2,725 participants with available baseline data for measures of the physical frailty syndrome, physiological dysregulation and adverse outcomes, who were followed up to 9 years (mean of 4.4) years for mortality. Follow up measures of functional aging and adverse health outcomes were available at 3–5 (mean 4.5) years after baseline interview for 1,298 participants. The study was approved by the Institutional Review Board (IRB) of National University of Singapore, and all participants provided written informed consent.

### Baseline Physiological Dysregulation

We derived log10-transformed values of the Mahalanobis distance (Dm) as proposed by Cohen and other investigators (8, 10, 17, 18) using available blood data at baseline for global PD. System-specific Dm measures for 6 domains of dysregulated function were derived using groups of blood biomarkers: (1) “metabolic”: fasting glucose, triglycerides, low-density lipoprotein (LDL)-cholesterol, high-density lipoprotein (HDL)-cholesterol, total cholesterol, cholesterol HDL-C ratio); (2) “renal”: estimated glomerular filtration rate (eGFR) for renal function; (3) “methylation”: homocysteine, folate, vitamin B12; (4) “oxygen transport”: hemoglobin, haematocrit, red blood cell distribution width, mean red corpuscular volume, mean corpuscular hemoglobin, mean corpuscular hemoglobin concentration; (5) “inflammation”: albumin, neutrophil-to-lymphocyte ratio, lymphocyte-to-monocyte ratio; and (6) “acute phase response and adhesion” (APRA): platelets, mean platelet volume, platelet to lymphocyte ratio, systemic immune-inflammation index. Values of blood folate, B12 and homocysteine were log10-transformed to approximate normality. The measurements of blood biomarkers were made using standard laboratory techniques by the National Research Laboratory (NRL), National University Hospital of Singapore.

Dm is assumed to be better represented by taking the overall average (centroid) of a “reference population” representing the “normal” physiological state in younger individuals (10, 29). Although a subset of younger healthier individuals may increase the strength of the dysregulation signal detected, validation analyses in previous studies suggests that a sample can be used as its own RP in most cases. This approach was adopted with a subsample of participants aged 55–64 years in this study, although we found that it provided negligibly enhanced acuity expected in sensitivity analyses.

### Baseline Physical Frailty

Frailty was assessed based on the 5 physical phenotype components proposed and validated by Fried and colleagues in the Cardiovascular Health Study (CHS) (5). (1) Involuntary or unintentional weight loss was defined as body mass index (BMI) below 18.5 kg/m2 and/or unintentional weight loss of above 10 pounds (4.5 kg) in the past 6 months. (2) Slowness was defined by the lowest quintile values (stratified for gender and height) in the average of 2 measurements of the 6-meter fast gait speed test. (3) Weakness was defined by the lowest quintile of a gender and BMI adjusted average value from 3 trials of the knee extension strength measured on the dominant knee. (4) Exhaustion was denoted as a score of <10 out of 15 on the vitality domain in the 12-Item Short Form Survey (SF-12). (5) Physical activities were assessed using self-reported time (in hours) spent daily doing light, moderate and vigorous activities. Low physical activity was defined by the lowest sex-specific quintile of the total amount of time spent on performing moderate to vigorous activities per week. A participant with 3 and more components was categorized as frail, 1–2 components as pre-frail, and none of the components as robust.

### Adverse Functional and Health Outcomes

At baseline and follow up, global cognitive function was assessed using a previously validated version of the Mini-Mental State Examination (MMSE) for use in local Singaporean older adults (30). Depressive symptoms were assessed using the 15-item Geriatric Depression Scale (GDS) which was also previously validated in local Singaporean older adults (31). Questionnaire interviews at baseline and follow up recorded the total number of prescription drugs used, the number of hospitalizations in the previous 1 year, and disability (the number of basic and instrumental activity of daily living requiring assistance) that were reported by the participant. Mortality follow up was performed by computerized matching search of the National Death Registry for registration and date of death by the National Disease Registry Office, between the date of initial interview up to 31 December 2016.

### Statistical Analysis

Data analysis was performed using IBM SPSS version 26. We examined the associations between PD or physical frailty (singly and jointly) with adverse health and functional outcomes, controlling for age, gender, and formal education level using Generalized Linear Models. The blood biomarker data were log-transformed as necessary to approach normality, and were divided by the standard deviation before calculating the Mahalanobis distance. The log10-transformed values of the Mahalanobis distance measures of PD were normally distributed and entered as continuous independent variables and frailty was entered as an ordinal categorical variable. For the dependent variables, linear regression was used to model MMSE score and the number of medications used, negative binomial regression was used to model depressive symptoms, number of IBADL disabilities, and number of hospitalizations, and Cox proportional hazard was used to model time-to-event data for mortality outcome. For the association of frailty and functional outcomes, F test was performed for MMSE score and number of medications used and Wald chi-squared test was performed for depressive symptoms, number of IBADL disabilities, and number of hospitalizations after adjustment of age, sex, and formal education level, or additionally PD. Sobel test was performed to determine the significance of the mediating effect of PD between the association of frailty and mortality. No adjustments were made for multiple comparisons due to the exploratory nature of the analyses. Statistically significant associations were flagged by *p* < 0.05.

## Results

### Descriptive Findings

The mean age of the participants was 66.6 (SD: 7.8) years, and 63% were women (Table 1). There were 48.1% (1,310) individuals who were robust, 46.8% (1,274) who were pre-frail, and 5.2% (141) who were frail. There were 180 deaths from 16,848 person-years of follow-up, giving an overall mortality rate of 10.7 deaths per 1,000 person-years.

**Table 1:** Baseline demographic, frailty, and physiological dysregulation profile of study participants (*n* = 2,725).

	**Mean** **±** **SD or** ***n*** **(percentage)**	
	**Baseline**	**Follow up**
Sample *N*	2,725	1,298
**Sex**		
Male	1,011 (37.1)	NA
Female	1,714 (62.9)	NA
Age (years)	66.6 ± 7.8	NA
**Formal education**		
No or primary education	1,687 (61.9)	NA
Secondary and above	1,038 (38.1)	NA
Frailty		NA
Robust	1,310 (48.1)	NA
Pre-frail	1,274 (46.8)	NA
Frail	141 (5.2)	NA
		NA
Physiological dysregulation^†^		NA
Metabolic function^†^	0.39 ± 0.43	NA
Renal function^†^	0.22 ± 0.23	NA
Methylation^†^	0.44 ± 0.30	NA
Oxygen transport^†^	0.72 ± 0.32	NA
Inflammation^†^	0.49 ± 0.27	NA
Adhesion^†^	0.58 ± 0.28	NA
Global^†^	1.24 ± 0.24	NA
**Functional aging and adverse outcomes**		
MMSE	27.81 ± 2.84	27.97 ± 2.51
GDS	0.74 ± 1.49	0.59 ± 1.10
No. of IADL or BADL	0.29 ± 1.12	0.23 ± 1.06
No. of medications used	2.26 ± 2.50	2.23 ± 2.39
No. of hospitalizations in past 1 year	0.06 ± 0.38	0.11 ± 0.57
No. of deaths	NA	180
Person-years of follow up	NA	16,848
Mortality per 1,000 person-years	NA	10.68

† *Data shown are log_10-_transformed Mahalanobis distance*.

*MMSE, Mini-mental State Examination; GDS, Geriatric Depression Scale; IADL, Instrumental Activity of Daily Living; BADL, basic Activities of Daily Living*.

*NA, Not applicable*.

### Global and System-Specific PDs

Correlations among system-specific PD scores with and without adjustment for age, sex and education, as shown in Supplementary Table 1, were either null or weakly positive, all below *r* = 0.29. Individual system-related PD were more strongly positive correlated with global PD, but all below *r* = 0.458. Global and system-related PD increased with age, and the quadratic term was significant for global, metabolic, methylation and inflammation, indicating an acceleration of PD with age (Supplementary Figure 1). Global and most system-related PD were significantly greater in men and those with less education.

### Global PD, Frailty, and Mortality

Global PD (as well as system-specific PDs) was significantly but weakly associated with frailty or pre-frailty (Table 2). As expected, physical frailty significantly predicted mortality risk (Table 3). In Cox regression adjusting for age, sex and education, pre-frailty-and-frailty predicted earlier mortality after follow up, with hazard ratio of 2.12 (95% CI = 1.51–3.00). Global PD also significantly predicted mortality (HR = 3.88, 95% CI = 2.15–6.98, *p* < 0.001) (Table 3). Adding global PD to the prediction model attenuated the association between pre-frailty-and-frailty and mortality, but the HR remained significant at 1.83 (95% CI = 1.22–2.73), while PD also remained significantly associated with mortality (HR = 3.06, 95% CI = 1.60–5.85). No significant interaction of PD and frailty was found. Sobel test showed that PD mediated the association between frailty and mortality (*Z* = 3.233, *p* = 0.001) (Table 3). Using receiver operating characteristic (ROC) analysis to assess the predictive accuracy of PD and physical frailty for mortality, each of them showed similar areas under the curve (AUC); PD: 0.640 (95% CI = 0.599–0.681); physical frailty: 0.621 (95% CI = 0.576–0.665). Combining them in the same model resulted in an increased predictive accuracy (AUC = 0.681; 95% CI = 0.638–0.724). The improvement of AUC after adding PD was statistically significant (DeLong's test: AUC difference = 0.0604, SE = 0.0130, 95% CI = 0.0349–0.0859, *Z* = 4.640, *p* < 0.001) (Table 4).

**Table 2:** Baseline associations of global and system-specific PD with physical frailty (*N* = 2,906).

	**Global**	**Metabolic**	**Renal**	**Methylation**	**Oxygen transport**	**Inflammation**	**Adhesion**
Robust (*N* = 1,310)	1.23 ± 0.01	0.37 ± 0.01	0.20 ± 0.01	0.41 ± 0.01	0.72 ± 0.01	0.48 ± 0.01	0.56 ± 0.01
Pre-frail (*N* = 1,274)	1.25 ± 0.01	0.43 ± 0.01^**^	0.23 ± 0.01^**^	0.45 ± 0.01^*^	0.73 ± 0.01	0.49 ± 0.01	0.59 ± 0.01^*^
Frail (*N* = 141)	1.33 ± 0.04^*^	0.46 ± 0.05	0.27 ± 0.03^*^	0.45 ± 0.01^*^	0.80 ± 0.04	0.54 ± 0.03	0.59 ± 0.03
*F* statistic	3.447	3.130	4.877	4.256	1.403	2.016	3.658
p (linear)	<0.001	0.002	<0.001	<0.001	0.161	0.044	<0.001
Partial eta-square	0.006	0.005	0.005	0.004	0.002	0.001	0.003

*Adjusted for age, sex, and education*.

*Data shown are log_10−_transformed Mahalanobis distance, mean ± SD*.

* *p < 0.05*,

** *p < 0.01*,

*** *p < 0.001 vs. robust*.

**Table 3:** Association of physical frailty and physiological dysregulation (PD) with mortality (*N* = 2,906).

	**Base models**			**Base model** **+** **global PD**			**Sobel test**	
	**HR**	**(95% CI)**	***P***	**HR**	**(95% CI)**	***P***	***Z***	***P***
Pre-frailty & frailty (vs. robust)	2.12	(1.51–3.00)	0.001	1.83	(1.22–2.73)	0.003		
Pre-frail (vs. robust)	1.96	(1.37–2.78)	<0.001	1.86	(1.24-2.80)	0.003		
Frail (vs. robust)	2.53	(1.50–4.28)	<0.001	1.59	(0.79–3.20)	0.199		
Global PD	3.88	(2.15–6.98)	<0.001	3.06	(1.60–5.85)	<0.001	3.233	0.001

*All models are adjusted for age, sex, education*.

*The interaction between PD and frailty was not significant*.

*NA: not applicable*.

**Table 4:** Receiver operating characteristic (ROC) analysis of the predictive accuracy of physiological dysregulation (PD) and physical frailty for mortality.

	**AUC**		**Delong's test**	
	**AUC**	**(95% CI)**	***Z***	***P***
Frailty	2.12	(1.51–3.00)		
PD	1.96	(1.37–2.78)		
Frailty + PD	2.53	(1.50–4.28)		
(Frailty + PD) vs. Frailty			4.640	<0.001

### Global PD, Frailty, and Other Adverse Outcomes

Physical frailty was also significantly associated with MMSE, GDS, disability, medications, and hospitalizations at baseline. It was significantly associated with all adverse outcomes except GDS and number of hospitalizations at follow up (Table 5). PD was significantly associated with MMSE, disability and medications, but not depression or hospitalization (Supplementary Table 2). Global PD attenuated the associations between frailty and all functional and adverse health outcomes at baseline (Table 5), but frailty remained associated with these outcomes. Likewise, physical frailty remained significantly associated with disability and the number of medications at follow up, but not with MMSE.

**Table 5:** Associations of physical frailty with adverse functional and health outcomes at baseline and follow up controlling for global physiological dysregulation^†^.

		**Base model (age, sex, and education)**					**Adjusted (base model** **+** **global PD)**				
**Model**	**Adverse functional**	**Robust**	**Pre-frail**	**Frail**	**Test statistic^†^**	***P***	**Robust**	**Pre-frail**	**Frail**	**Test statistic^†^**	***P***
	**health outcomes**										
Baseline	*N* = 2,725	1,310	1,274	141							
	MMSE	28.36 ± 0.07	28.04 ± 0.08^**^	26.50 ± 0.28^***^^###^	22.721	<0.001	28.36 ± 0.09	28.08 ± 0.10	26.74 ± 0.40^***^^###^	8.595	<0.001
	GDS	0.51 ± 0.04	0.76 ± 0.04^***^	1.75 ± 0.16^***^^###^	110.261	<0.001	0.51 ± 0.05	0.86 ± 0.06^***^	1.64 ± 0.23^***^^###^	63.001	<0.001
	No. of IADL or BADL	0.13 ± 0.03	0.31 ± 0.03^***^	1.14 ± 0.11^***^^###^	190.591	<0.001	0.12 ± 0.03	0.31 ± 0.04^***^	1.16 ± 0.15^***^^###^	108.022	<0.001
	No. of medications	2.01 ± 0.07	2.34 ± 0.07^**^	3.10 ± 0.27^***^^#^	24.491	<0.001	2.09 ± 0.09	2.36 ± 0.10	2.96 ± 0.39^**^^##^	9.871	0.007
	No. of hospitalizations	0.04 ± 0.01	0.07 ± 0.01	0.17 ± 0.04^**^	33.383	<0.001	0.03 ± 0.01	0.05 ± 0.01	0.26 ± 0.04^***^^###^	20.851	<0.001
Follow up	*N* = 1,298	670	583	45							
	MMSE	28.32 ± 0.12	28.05 ± 0.12	27.46 ± 0.37^*^	4.011	0.018	28.33 ± 0.16	28.16 ± 0.18	28.16 ± 0.66	0.436	0.647
	GDS	0.54 ± 0.05	0.55 ± 0.06	0.84 ± 0.17	2.950	0.229	0.67 ± 0.08	0.64 ± 0.09	0.50 ± 0.32	0.502	0.778
	No. of IADL or BADL	0.15 ± 0.05	0.26 ± 0.05	0.75 ± 0.16^***^^###^	27.622	<0.001	0.20 ± 0.07	0.22 ± 0.08	1.10 ± 0.28^**^^##^	23.282	<0.001
	No. of medications	1.99 ± 0.11	2.31 ± 0.12^*^	3.92 ± 0.35^***^^###^	16.378	<0.001	2.23 ± 0.15	2.26 ± 0.17	3.93 ± 0.62^*^^#^	3.707	0.025
	No. of hospitalizations	0.11 ± 0.03	0.11 ± 0.03	0.16 ± 0.09	0.647	0.724	0.12 ± 0.04	0.07 ± 0.04	0.17 ± 0.16	4.170	0.124

*Test statistic*

† *: F (MMSE and number of medications); Wald chi-squared (GDS, no. of IBADL, no. of hospitalizations)*.

* *p < 0.05*,

** *p < 0.01*,

*** *p < 0.001 vs. robust*.

# *p < 0.05*,

## *p < 0.01*,

### *p < 0.001 vs. pre-frailty*.

### System-Specific PD, Frailty, and Adverse Outcomes

These were explored in secondary analyses. Except for oxygenation, system-specific PDs were significantly associated with frailty or pre-frailty (Table 2). System-specific PDs showed mixed associations with adverse outcomes (Supplementary Table 2). System-specific PDs significantly predicted mortality, but with smaller effect sizes. They were all significantly associated with the number of medications used at baseline. All PDs except methylation and adhesion PDs were associated with number of IBADL impairments. Metabolic function, renal function, and inflammation PD were significantly associated with MMSE score. Only methylation PD was significantly associated with depression, and only oxygen transport was significantly associated with hospitalization.

## Discussion

The physical frailty phenotype is widely recognized as a clinical frailty syndrome and multi-system physiological dysregulation may be appropriately viewed as its biological substrate, or endo-phenotype. Our study findings contribute additional insights into the role and contribution of PD through its co-occurrence with frailty and the known impact of frailty on adverse outcomes. As shown in this and other studies (20, 32), PD and frailty have independent effects in predicting mortality and adverse functional and health outcomes. Although the deleterious effects of frailty were attenuated by PD in the model, we found that frailty remained predictive of mortality and other adverse outcomes. As a multi-dimensional construct, frailty has effects which can be explained by the contributions of other known factors particularly social and environmental ones, including limited social support and access to healthcare which are known determinants of frailty and adverse outcomes. On the other hand, PD was predictive of mortality as well as cognitive function, disability, and polypharmacy, but not depression and hospitalization. This suggests that PD accounts substantially for the emergence of cognitive and physical functional impairment associated with frailty. As a biological construct, it will have weaker contributions to distal health outcomes such as depression or hospitalization which are strongly determined by social factors such as social isolation and access to health and social services. Taken together, our observations fit a developmental model in which physiological dysregulation arising from the aging process perpetuates a deleterious course of clinical frailty. At the tissue, organ and system biology levels, physiological dysregulation may be responsible for the emergence of physical frailty, but beyond that, psychological, social, environmental factors (33) are likely to shape variable trajectories of frailty progression resulting in adverse health outcomes culminating in death (33).

In this study, we used the physical frailty measure to define the clinical phenotype of frailty, and the Dm measure of physiological dysregulation (PD-Dm) to define the frailty substrate or endo-phenotype. Other studies have used different approaches to define and measure frailty and its endo-phenotype. An alternative approach is to use the Frailty Index (FI) based on the “cumulated deficits” model. The FI is expressed as the ratio of the health deficits present in a person to the total number of potential health deficits evaluated, and has been used as an alternative to the physical frailty phenotype in many studies. It has also been used to variously represent biological age, “allostatic load,” and physiological dysregulation. The FI health deficits are broadly inclusive of any symptoms, signs, diseases, functional impairments, as well as laboratory abnormalities. Together, they represent a clinical phenotype of frailty (robust, pre-frail, or frail) when dichotomized or polychotomized using the appropriate cutoffs. As such, it could thus be viewed to incorporate both the clinical (exo-phenotype) and the endo-phenotype of frailty. A blood biomarker-based frailty index (FI-B) of the frailty endo-phenotype has been evaluated for its utility for the first time using data from the Newcastle 85+ Study (34). The study found FI-B to be a significant predictor of mortality, but with less predictive accuracy than a FI based on clinical deficits (Clinical Deficits Frailty Index; FI-CD). Like the Dm measure of physiological dysregulation (PD-Dm) used in our study, the FI-B was found to add to the ability of FI-CD to predict adverse outcomes, especially those without detectable clinical frailty deficits. The FI-B is conceptually close to the Dm measure of PD as a measure of frailty endophenotype, but uses a different computational algorithm (34). Although based on different algorithmic approaches, there is agreement that both the PD-Dm and FI-B alike appear to tap on the same multisystem level biological substrate of clinical frailty. However, PD may have advantages over FI-B in some aspects. As a truly continuous variable, PD is statistically superior than FI-B, and is not constrained by the requirement for a minimum number of (at least 30 or 40) variables. Furthermore, system-specific PD-Dm can also be calculated with a handful of variables to supplement global PD-Dm, while deriving a system-specific FI-B measure is obviously cumbersome, and only a global measure of FI-B has so far been validated for use. Further studies may perhaps throw more light on the relative advantages and disadvantages of these different approaches.

Our findings suggest that viewing and measuring frailty as biological and clinical constructs together has clinical utility. The measurement of clinical frailty in older patients is already widely recognized to be clinically useful in predicting future risks of adverse outcomes. Consonant with this, physical frailty was found in this study to predict mortality, as well as other adverse outcomes. However, the predictive accuracy of clinical frailty, either by the physical frailty phenotype or the frailty index, remain limited. As shown in this study, as well as previous studies, the area under the curve for mortality prediction are ~0.60 or less (35, 36). Our study shows that the predictive accuracy of clinical frailty is increased by the addition of the Dm measure of PD. Extant evidence suggest that multi-system PD predicts the onset of the clinical frailty syndrome (15, 16), although this is not shown by the data in this study. Our study shows that the co-occurrence of global PD with prevalent physical frailty is more than just co-incidental. It is pertinent to note that not all individuals with high PD are frail, neither do all frail individuals have high PD. Adding a measure of global PD provides additional information that increase the power to predict adverse health outcomes. Hence a frail older person with high global PD will have a greater expectation of subsequent adverse health events than one without high PD. Conversely, in a robust older person, high PD provides a preclinical indication of increased likelihood of becoming frail, and/or increased risk of disease, functional aging morbidity and adverse health events.

The utility of the Dm measure of global PD is easily portable from research to the clinical setting, given present day data-driven and digital health standards of primary and hospital practice. Most health care facilities are high volume repositories of massive amount of clinical information including patients' blood tests and physical examinations permitting algorithmic processing of the data to generate automatically Dm values of physiological dysregulation. Further studies should validate the clinical utility of this approach. Furthermore, the measures of system-specific dysregulation may potentially provide fine-grained information that permit a deeper understanding and characterization of the pre-clinical frailty endo-phenotype. For example, its feasibility has recently been demonstrated by a recent study that identified a set of plasma metabolites (organic acids/derivatives or lipids/lipid-like molecules) associated with frailty that attenuated a substantial portion of the frailty-associated risk of death (37). However, more studies are needed.

### System-Specific PD

Our secondary analyses of data suggest that it may also be useful to consider the roles and contributions of system-specific dysregulation. In agreement with previous studies, our study found that they are only weakly correlated or uncorrelated with one another, but individually are more strongly correlated with global PD, suggesting the amplifying effect of semi-independent dysregulated processes interacting in feedback fashion with each other (19, 21). One previous study has reported that multiple system-specific dysregulation scores are more strongly associated with frailty and mortality, while their associations are more ambiguous for cardiovascular disease and not significant for cancer (19). We observe that some system-specific dysregulations are associated with frailty and predict mortality risk, disabilities and polypharmacy, but not others. A small number of system-specific dysregulations (metabolic, renal, and inflammation) appear to be specifically associated with cognition; only methylation was associated with depression, and only oxygenation (red blood cells and hemoglobin) was associated with hospitalization. Hence there appears to be a heterogeneity of associations of system-specific dysregulations with different adverse outcomes. A comprehensive examination is beyond the scope for discussion in this paper, even with the non-exhaustive set of system-specific PDs based on available blood biomarkers in this study. We could highlight a singular example: the association between “methylation” dysregulation and depression is plausible and meaningful, given the central role of folate, B12 and homocysteine in the methylation cycle responsible for donating methyl groups in the synthesis of monoamine neurotransmitters (serotonin and dopamine).

Finally, a number of limitations in the analyses should be noted. One is that PD and frailty were measured only at baseline. As frailty and PD are not invariant over time, some positive associations observed at baseline in cross-sectional analyses were not replicated in longitudinal analyses, and time-varying strengths or effects were not taken into account. A strength of the present study is the prospective follow up of the participants over a mean of 4.5 years for adverse outcomes. However, health deterioration may be different during the 4.5 years for participants with different age, e.g., those at 55 and at 85 years. However, in this relatively young aging cohort, only 2% were aged 85 and above, and 14% were aged 75 and above. The standardized questionnaires used to measure cognitive function and depressive symptoms, namely MMSE and GDS, show low inter-individual variation and maybe a ceiling effect. This could attenuate the estimate of association associated with PD-Dm, and limit its ability to detect some significant effects. Our analyses did not include data on clinical factors such as multi-morbidity, and psychological, social and environmental factors which may shape variable trajectories of frailty progression and adverse health outcomes culminating in death, and therefore limit the scope of our inquiry.

## Conclusions

In conclusion, our study suggests that co-existing physiological dysregulation and physical frailty independently predict mortality and functional and health outcomes in older adults, with increased predictive accuracy when combined. PD appears to be a valid representation of a biological endo-phenotype of frailty. Further studies could investigate the potential utility of similar subclinical measures of frailty.

## Data Availability Statement

The raw data supporting the conclusions of this article will be made available by the authors, without undue reservation.

## Ethics Statement

The studies involving human participants were reviewed and approved by the Institutional Review Board (IRB) of National University of Singapore. The patients/participants provided their written informed consent to participate in this study.

## Author Contributions

YL did the statistical analyses and drafted the manuscript. TPN designed the study and directed the data collection as the principal investigator of the SLAS study. All authors contributed to the study concept and design, to the interpretation of data, to the critical review of the manuscript, and approved the final version.

## Funding

The study was supported by Biomedical Research Council (BMRC/08/1/21/19/567) and the National Medical Research Council (NMRC/1108/2007; NMRC/CIRG/1409/2014).

## Conflict of Interest

The authors declare that the research was conducted in the absence of any commercial or financial relationships that could be construed as a potential conflict of interest.

## Publisher's Note

All claims expressed in this article are solely those of the authors and do not necessarily represent those of their affiliated organizations, or those of the publisher, the editors and the reviewers. Any product that may be evaluated in this article, or claim that may be made by its manufacturer, is not guaranteed or endorsed by the publisher.
