# Supplementary material for: Physiological Dysregulation, Frailty, and Impacts on Adverse Health and Functional Outcomes
Source: Front Med (Lausanne). 2021 Oct 22;8:751022. doi: 10.3389/fmed.2021.751022 (PMC8569246; doi:10.3389/fmed.2021.751022)
Supplement: Supplementary file 1 [file Data_Sheet_1.docx]

Supplementary Material

|  |
| --- |

**Supplementary Table S1**. Correlations among system and global PD, controlling for age, sex and education

|  | Unadjusted | | | | | | |  | Adjusted for age, sex and education | | | | | | |
| --- | --- | --- | --- | --- | --- | --- | --- | --- | --- | --- | --- | --- | --- | --- | --- |
|  | Metabolic | Renal | Methylation | Oxygenation | Inflammation | Adhesion | Global |  | Metabolic | Renal | Methylation | Oxygenation | Inflammation | Adhesion | Global |
| Metabolic | 1 | .072^**^ | 0.019 | 0.075^**^ | 0.061^**^ | 0.070^**^ | 0.426^**^ |  | 1.000 | 0.088*** | 0.032 | 0.042 | 0.087** | 0.050* | 0.432*** |
| Renal |  | 1 | .241^**^ | 0.055^**^ | 0.026 | 0.061^**^ | 0.298^**^ |  |  | 1.000 | 0.227*** | 0.063* | 0.036 | 0.077** | 0.301*** |
| Methylation |  |  | 1 | 0.023 | -0.002 | 0.073^**^ | 0.403^**^ |  |  |  | 1.000 | 0.029 | -0.007 | 0.066** | 0.408*** |
| Oxygenation |  |  |  | 1 | 0.029 | 0.055^**^ | 0.458^**^ |  |  |  |  | 1.000 | -0.016 | -0.005 | 0.445*** |
| Inflammation |  |  |  |  | 1 | 0.304^**^ | 0.392^**^ |  |  |  |  |  | 1.000 | 0.291*** | 0.398*** |
| Adhesion |  |  |  |  |  | 1 | 0.434^**^ |  |  |  |  |  |  | 1.000 | 0.443*** |
| Global |  |  |  |  |  |  | 1 |  |  |  |  |  |  |  | 1.000 |

* p<0.05, ** p<0.01, *** p<0.001 (2-tailed).

|  |
| --- |

| Supplementary Table S2. Associations of baseline physiological dysregulation with adverse functional and health outcomes at baseline and follow up | | | | | | | | | | | |
| --- | --- | --- | --- | --- | --- | --- | --- | --- | --- | --- | --- |
| Dependent | Independent variable | *Adverse health outcomes at baseline* | | | |  | *Adverse health outcomes at follow up* | | | | |
| Adverse health outcome | Baseline PD | B | (95%CI) | β | *P* |  | B | (95%CI) | β | *P* | |
|  |  |  |  |  |  |  |  |  |  |  | |
| MMSE cognition | Global | -1.249 | -1.764, -0.730 | -0.107 | <0.001 |  | 0.027 | -0.779, 0.834 | 0.003 | 0.947 | |
|  | Metabolic function | -0.409 | -0.626, -0.191 | -0.062 | <0.001 |  | -0.155 | -0.488, 0.179 | -0.026 | 0.362 | |
|  | Renal function | -1.040 | -1.438, -0.642 | -0.086 | <0.001 |  | -0.756 | -1.391, -0.120 | -0.067 | 0.020 | |
|  | Methylation | -0.317 | -0.722, 0.089 | -0.034 | 0.126 |  | 0.270 | -0.395, 0.935 | 0.032 | 0.426 | |
|  | Oxygen transport | -0.173 | -0.467, 0.121 | -0.020 | 0.248 |  | 0.014 | -0.470, 0.497 | 0.002 | 0.956 | |
|  | Inflammation | -0.469 | -0.814, -0.124 | -0.045 | 0.008 |  | -0.548 | -1.081, -0.015 | -0.058 | 0.044 | |
|  | Adhesion | -0.256 | -0.589, 0.077 | -0.026 | 0.132 |  | -0.193 | -0.695, 0.309 | -0.022 | 0.450 | |
|  |  |  |  |  |  |  |  |  |  |  | |
| GDS depression | Global | 0.098 | -0.216, 0.412 | 0.015 | 0.541 |  | 0.027 | -0.342, 0.397 | 0.006 | 0.884 | |
|  | Metabolic function | 0.011 | -0.112, 0.134 | 0.003 | 0.859 |  | -0.081 | -0.221, 0.059 | -0.031 | 0.258 | |
|  | Renal function | 0.106 | -0.127, 0.339 | 0.017 | 0.373 |  | -0.185 | -0.449, 0.079 | -0.037 | 0.170 | |
|  | Methylation | 0.378 | 0.125, 0631 | 0.073 | 0.003 |  | 0.172 | -0.135, 0.479 | 0.041 | 0.271 | |
|  | Oxygen transport | 0.011 | -0.159, 0.181 | 0.002 | 0.904 |  | 0.129 | -0.066, 0.324 | 0.036 | 0.194 | |
|  | Inflammation | 0.166 | -0.032, 0.364 | 0.031 | 0.100 |  | 0.104 | -0.116, 0.324 | 0.025 | 0.353 | |
|  | Adhesion | 0.005 | -0.187, 0.197 | 0.001 | 0.957 |  | 0.080 | -0.130, 0.289 | 0.020 | 0.456 | |
|  |  |  |  |  |  |  |  |  |  |  |  |
| Number of IBADL | Global | 0.269 | 0.063, 0.475 | 0.063 | 0.011 |  | 0.266 | -0.073, 0.604 | 0.058 | 0.123 |  |
| Impairments | Metabolic function | 0.092 | 0.000, 0.184 | 0.036 | 0.050 |  | -0.012 | -0.147, 0.122 | -0.005 | 0.856 |  |
|  | Renal function | 0.313 | 0.144, 0.482 | 0.066 | <0.001 |  | 0.063 | -0.190, 0.316 | 0.013 | 0.625 |  |
|  | Methylation | 0.054 | -0.115, 0.223 | 0.016 | 0.509 |  | -0.153 | -0.431, 0.126 | -0.040 | 0.281 |  |
|  | Oxygen transport | 0.178 | 0.053, 0.303 | 0.051 | 0.005 |  | 0.130 | -0.058, 0.317 | 0.037 | 0.175 |  |
|  | Inflammation | 0.206 | 0.061, 0.351 | 0.050 | 0.005 |  | 0.336 | 0.126, 0.546 | 0.085 | 0.002 |  |
|  | Adhesion | 0.132 | -0.009, 0.273 | 0.033 | 0.068 |  | 0.336 | 0.136, 0.537 | 0.089 | 0.001 |  |
|  |  |  |  |  |  |  |  |  |  |  |  |
| Number of medications used | Global | 1.830 | 1.340, 2,320 | 0.174 | <0.001 |  | 0.704 | -0.046, 1.453 | 0.069 | 0.066 |  |
|  | Metabolic function | 0.806 | 0.604, 1.008 | 0.139 | <0.001 |  | 0.706 | 0.405, 1.007 | 0.124 | <0.001 |  |
|  | Renal function | 1.310 | 0.940, 1.680 | 0.123 | <0.001 |  | 0.796 | 0.225, 1.366 | 0.074 | 0.006 |  |
|  | Methylation | 1.464 | 1.080, 1.848 | 0.176 | <0.001 |  | 1.254 | 0.634, 1.874 | 0.146 | <0.001 |  |
|  | Oxygen transport | 0.433 | 0.159, 0.707 | 0.056 | 0.002 |  | 0.514 | 0.093, 0.934 | 0.065 | 0.017 |  |
|  | Inflammation | 0.496 | 0.177, 0.815 | 0.054 | 0.002 |  | 0.118 | -0.358, 0.593 | 0.013 | 0.627 |  |
|  | Adhesion | 0.457 | 0.145, 0.769 | 0.051 | 0.004 |  | 0.328 | -0.125, 0.781 | 0.039 | 0.156 |  |
|  |  |  |  |  |  |  |  |  |  |  |  |
| Number of hospitalizations in past one year | Global | 0.028 | -0.027, 0.83 | 0.025 | 0.319 |  | -0.040 | -0.222, 0.142 | -0.016 | 0.669 |  |
|  | Metabolic function | 0.018 | -0.013, 0.049 | 0.021 | 0.259 |  | 0.076 | 0.003, 0.149 | 0.056 | 0.040 |  |
|  | Renal function | 0.032 | -0.027, 0.091 | 0.020 | 0.277 |  | 0.049 | -0.089, 0.186 | 0.019 | 0.489 |  |
|  | Methylation | 0.001 | -0.044, 0.046 | 0.001 | 0.959 |  | 0.011 | -0.139, 0.162 | 0.006 | 0.881 |  |
|  | Oxygen transport | 0.041 | 0.001, 0.082 | 0.038 | 0.048 |  | 0.028 | -0.072, 0.128 | 0.015 | 0.584 |  |
|  | Inflammation | 0.039 | -0.010, 0.088 | 0.029 | 0.123 |  | 0.048 | -0.065, 0.160 | 0.023 | 0.405 |  |
|  | Adhesion | 0.021 | -0.028, 0.070 | 0.016 | 0.382 |  | -0.029 | -0.136, 0.079 | -0.014 | 0.602 |  |
|  |  |  |  |  |  |  |  |  |  |  |  |
| Values of independent variables are log_10-_transformed Mahalanobis distance.  Data are adjusted for age, gender, and formal education level.  B is the unstandardized regression coefficient which shows the slope of the line. β is the standardized regression coefficient which renders comparison among different models.  MMSE = Mini-mental State Examination; GDS = Geriatric Depression Scale; IADL = Instrumental Activity of Daily Living; BADL = basic Activities of Daily Living. | | | | | | | | | | |  |


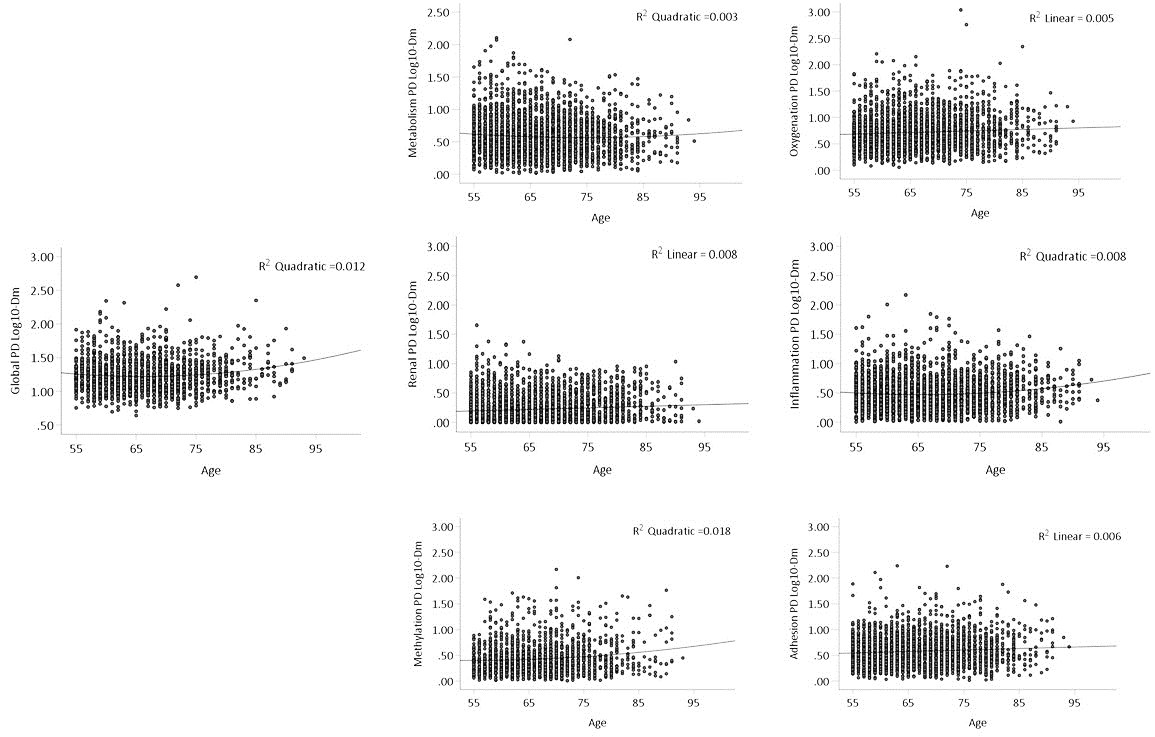


Figure S1. Correlation of global and system-related physiological dysfunction with age.
